# Microfluidic Synthesis of -NH_2_- and -COOH-Functionalized Magnetite Nanoparticles

**DOI:** 10.3390/nano12183160

**Published:** 2022-09-12

**Authors:** Cristina Chircov, Alexandra Cătălina Bîrcă, Bogdan Stefan Vasile, Ovidiu-Cristian Oprea, Keng-Shiang Huang, Alexandru Mihai Grumezescu

**Affiliations:** 1 Department of Science and Engineering of Oxide Materials and Nanomaterials, University Politehnica of Bucharest, 011061 Bucharest, Romania; 2 National Research Center for Micro and Nanomaterials, University Politehnica of Bucharest, 060042 Bucharest, Romania; 3 National Research Center for Food Safety, University Politehnica of Bucharest, 060042 Bucharest, Romania; 4 Department of Inorganic Chemistry, Physical Chemistry and Electrochemistry, University Politehnica of Bucharest, 1-7 Polizu Street, 011061 Bucharest, Romania; 5 The School of Chinese Medicine for Post-Baccalaureate, I-Shou University, Kaohsiung 840301, Taiwan; 6 Research Institute of the University of Bucharest—ICUB, University of Bucharest, 050657 Bucharest, Romania; 7 Academy of Romanian Scientists, 54 Splaiul Independentei, 050045 Bucharest, Romania

**Keywords:** microfluidics, lab-on-chip, magnetite nanoparticles, nanoparticle functionalization, amino, carboxyl

## Abstract

Microfluidics has emerged as a promising alternative for the synthesis of nanoparticles, which ensures precise control over the synthesis parameters, high uniformity, reproducibility, and ease of integration. Therefore, the present study investigated a one-step synthesis and functionalization of magnetite nanoparticles (MNPs) using sulfanilic acid (SA) and 4-sulfobenzoic acid (SBA). The flows of both the precursor and precipitating/functionalization solutions were varied in order to ensure the optimal parameters. The obtained nanoparticles were characterized through dynamic light scattering (DLS) and zeta potential, X-ray diffraction (XRD), selected area electron diffraction (SAED), transmission electron microscopy (TEM) and high-resolution TEM (HR-TEM), Fourier transform infrared spectroscopy (FT-IR), thermogravimetry and differential scanning calorimetry (TG-DSC), and vibrating sample magnetometry (VSM). The results demonstrated the successful synthesis of magnetite as the unique mineralogical phase, as well as the functionalization of the nanoparticles. Furthermore, the possibility to control the crystallinity, size, shape, and functionalization degree by varying the synthesis parameters was further confirmed. In this manner, this study validated the potential of the microfluidic platform to develop functionalized MNPs, which are suitable for biomedical and pharmaceutical applications.

## 1. Introduction

Magnetite is a naturally occurring iron oxide that is characterized by an inverse spinel structure, which crystallizes in the cubic system [1,2,3]. Magnetite represents, by far, one of the most intensively studied and applied types of nanoparticles within a variety of fields, such as biomedicine and the pharmaceutical, cosmetic, and food industries [4,5,6,7,8,9,10].

While most of the available studies investigate the efficiency of magnetite nanoparticles (MNPs) as nanocarriers for the delivery of bioactive molecules [11,12], they are generally synthesized through the co-precipitation method. Although it is one of most facile and cost-efficient methods, the obtained nanoparticles are usually characterized by a series of disadvantages in regard to their tunability and uniformity [10,13]. Specifically, the co-precipitation method does not allow for the precise control and modulation of the nanoparticle size and size distribution, shape, polydispersity, and crystallinity [14].

Considering that the biomedical and pharmaceutical fields require highly limited and precise intervals of size and shape, the synthesis of MNPs must ensure strict control over these characteristics in order to guarantee adequate biocompatibility, biodistribution, pharmacokinetics, and body clearance [15]. Thus, the focus should shift towards the improvement and development of synthesis methods that could provide more standardized parameters of the obtained nanoparticles.

In this context, microfluidic methods have emerged as a promising alternative for the synthesis of nanoparticles. Since they allow for the precise manipulation of fluids through the use of channels and chambers with sizes of tens to hundreds of micrometers, microfluidic technologies have the potential to outperform conventional synthesis methods. Specifically, they enable the precise, multistep control of the synthesis parameters, product uniformity, reproducibility, high throughput, and ease of integration [16,17]. The field of nanoparticle microfluidics is especially evolving in the area of magnetite synthesis [18]. To this date, there have been many studies reporting the synthesis of MNPs through microsized channels, demonstrating significantly narrow size distribution [19,20,21,22,23,24,25,26]. The available reports investigate the influence of microchannel geometry (e.g., X- and Y-junction) [19,21,23,25,26] and size [21], reagent solution concentration [1,20], and reaction solution flows [1] on the morphology of the nanoparticles. Moreover, the microfluidic synthesis of MNPs coated with various polymers in order to improve stability and biocompatibility have also been reported [22,23,26]. To the best of our knowledge, this is the first report on the one-step synthesis and functionalization of the MNPs.

In this context, the aim of this study was to investigate the influence of the synthesis parameters, namely the flow of the solutions and the concentration of the functionalization agent, on the properties of one-step -NH_2_-/-COOH-functionalized MNPs synthesized through a lab-on-chip (LoC) device. Thus, the obtained results could greatly benefit the process of drug delivery system development through superior control over the features of the nanocarrier and the amount of drug added.

## 2. Materials and Methods

### 2.1. Materials

Ferrous sulfate heptahydrate (FeSO_4_·7H_2_O), ferric chloride (FeCl_3_·6H_2_O), ammonium hydroxide (NH_4_OH), and 4-sulfobenzoic acid potassium salt (SBA—KO_3_SC_6_H_4_CO_2_H) were purchased from Sigma-Aldrich Merck (Darmstadt, Germany). Sulfanilic acid (SA—C_6_H_7_NO_3_S) was purchased from Carl Roth (Karlsruhe, Baden-Württemberg, Germany). All chemicals were of analytical purity and used with no further purification.

### 2.2. Methods

#### 2.2.1. LoC Device Design and Fabrication

The LoC device was designed using the RDWorksV8 software (Informer Technologies, Inc., Los Angeles, CA, USA), and it was based on the geometry employed in previous research papers (Figures S1 and S2) [1,26,27,28,29,30,31,32,33]. The LoC device contains three poly(methyl methacrylate) (PMMA) layers of the same size, provided with twenty overlayed orifices for placing M4 bolts (4.0 mm in diameter, 0.5 mm pitch, and tightened by a force of 2 Nm) for fixating the microfluidic chip. The top layer comprises three reagent inlets, the middle layer, the cross-junction channel (Figure S2), and the bottom layer, the outlet for reaction products. For each inlet and outlet, an additional PMMA pad was used for ensuring the fastening of the polyvinyl chloride (PVC) tubes (2.54 mm outer diameter). The polymeric components were fabricated using the 1610 Pro laser cutting machine (RUBIQ CNC, Bacău, Romania).

#### 2.2.2. Stock Solution Preparation

The stock precursor solution was prepared by dissolving FeSO_4_·7H_2_O and FeCl_3_·6H_2_O in ultrapure water at the 1:2 molar ratio and a final concentration of 1%. The precipitating agent solution was prepared with NH_4_OH at a 1 M concentration. Concomitantly, the solutions for the functionalization of the nanoparticles were prepared by dissolving SA for the -NH_2_ functionalization and SBA for the -COOH functionalization at three different concentrations (1, 3, and 5%) in the NH_4_OH 1 M solution. The concentrations of the functionalization agents refer to the percentage of SA or SBA calculated for the total amount of MNPs that would be obtained using the specified mass of iron precursors.

#### 2.2.3. Magnetite Nanoparticle Synthesis

The pristine and -NH_2_/-COOH-functionalized MNPs were obtained through the co-precipitation of the iron ions at the junction with the alkaline solution. The solutions were simultaneously introduced into the LoC device using a peristaltic pump with four individual channels. Specifically, the precursor solution was injected through the central inlet at 10 flows, while the precipitating/functionalization solution was introduced through the side inlets at two flows, namely 15 and 25 rpm for each tube, in order to achieve a final flow of 30 and 50 rpm, respectively. The so-obtained nanoparticles were dripped from the outlet, washed with ultrapure water until a neutral pH was achieved, and dried at 40 °C for 48 h. Table 1 summarizes the flows used for each type of the synthesized nanoparticles.

#### 2.2.4. Physicochemical Characterization of the Nanoparticles

##### Dynamic Light Scattering (DLS) and Zeta Potential

Using the ultrasonic bath, the nanoparticles were homogenously dispersed in deionized water (~6.9 pH) at a concentration of 0.3 mg/mL and introduced into the measurement cell of the DelsaMax Pro equipment (Backman Coulter, Brea, CA, USA). Each measurement involved a number of three acquisitions and a time of 5 s per acquisition. For each sample, measurements were performed in triplicate.

##### X-ray Diffraction (XRD) Coupled with Rietveld Refinement

The diffractograms were acquired using CuKα radiation of λ = 1.541874 Å within the PANalytical Empyrean diffractometer (PANalytical, Almelo, the Netherlands), equipped with a parallel-plate collimator on the PIXcel3D detector. The 2θ angle ranged between 20 and 80°, with the incidence angle of 0.5°, step size of 0.0256°, and time per step of 255 s. The crystallite size, unit cell parameters, and nanoparticle crystallinity were determined through the Rietveld refinement algorithm, using the HighScore Plus software (version 3.0, PANalytical, Almelo, the Netherlands).

##### Transmission Electron Microscopy (TEM), High-Resolution TEM (HR-TEM), Selected Area Electron Diffraction (SAED)

An amount of 10 µL of the suspension obtained by dispersing a small sample amount of the nanoparticles into deionized water was placed on a 400-mesh lacey carbon-coated copper grid at room temperature. The nanoparticles were characterized using a high-resolution 80–200 TITAN THEMIS transmission microscope (purchased from FEI, Hillsboro, OR, USA), equipped with an image corrector and EDXS detector in the column and operated at a 200 kV voltage in transmission mode. Particle size distribution was assessed by measuring 100 nanoparticles within the TEM images, using the ImageJ software (University of Wisconsin, Madison, WI, USA).

##### Fourier Transform Infrared Spectroscopy (FT-IR)

A Thermo Scientific Nicolet iS50 (Thermo Fischer Scientific, Waltham, MA, USA) spectrometer, with a liquid nitrogen-cooled mercury cadmium telluride detector, was used for the acquisition of the IR spectra. Measurements were performed at room temperature, in the range of 4000–400 cm^−1^ and at a resolution of 4 cm^−1^, using the attenuated total reflectance (ATR) mode. For each sample, the OmnicPicta software (version 8.2 Thermo Nicolet, Thermo Fischer Scientific, Waltham, MA, USA) was used for the co-adding and processing of the 64 scans acquired.

##### Thermogravimetry and Differential Scanning Calorimetry (TG-DSC)

The samples were placed in an alumina crucible and heated in a dynamic air atmosphere of 50 mL/min from 20 to 900 °C, at a heating rate of 10 K/min. The thermogravimetric analysis was performed using the STA TG/DSC Netzsch Jupiter 449 F3 equipment (Selb, Germany).

##### Vibrating Sample Magnetometry (VSM)

The magnetic properties of the MNPs were determined at room temperature (25 °C), using the Cryogen-free Vibrating Sample Magnetometer (VersaLabTM 3T, Westerville, OH, USA). The range of the applied magnetic field was between −10 and +10 kOe and vice versa, with a step rate of 10 Oe/s.

## 3. Results

The present study aimed to develop a series of -NH_2_-/-COOH-functionalized MNPs using an LoC device. The synthesis parameters were varied in regard to the concentration of the functionalization agent and the flows within the microfluidic platform. Therefore, the initial step of the study was based on the optimization of the flow for increasing the uniformity of the functionalization process.

In this context, the first physicochemical characterization method employed after synthesis was DLS and zeta potential in order to determine the optimal flows that could ensure the successful functionalization of the MNPs. The hydrodynamic diameters and zeta potential values for all the obtained nanoparticles are shown in Figures S3–S6. The results show no significant differences between the hydrodynamic diameter values of the -NH_2_- and -COOH-functionalized MNPs, which could mean that the type of the functionalization agent does not influence the agglomeration tendency of the nanoparticles. The increase in the concentration of the functionalization agent leads to lower hydrodynamic diameters, due to the presence of the amino/carboxyl moieties that prevent the agglomeration of the MNPs. The zeta potential measurements confirm these observations, as higher hydrodynamic diameter values are associated with lower zeta potentials. Such behavior is expected, as increased zeta potential values are a consequence of higher amounts of charges on the surface of the nanoparticles, which lead the repulsive forces to exceeding the attractive forces [34,35]. Since all measurements were performed in deionized water, thus in a neutral pH, the -NH_2_ and -COOH groups were not ionized to -NH_3_^+^ and -COO-, respectively. The lack of uniformity within all samples might be caused by the effects of the solution flows on the size distributions of the nanoparticles. Specifically, flows of the precipitating/functionalization solution higher than the flow of the precursor solution lead to the formation of smaller nucleation centers, due to the high turbulences generated at the cross-junction and, consequently, to reduced variations in the nanoparticle sizes. In this case, the optimal ratio for obtaining uniform functionalization varies between 1.66:1 and 1:1. By contrast, lower flow values restrict the optimal flows at the ratio of 1:1, due to limited turbulences formed at the cross-junction that cause nucleation centers of varying sizes.

**Figure 1:**
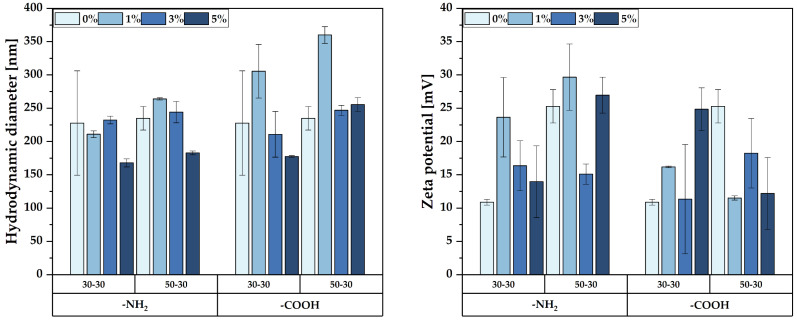
The hydrodynamic diameter and zeta potential values of the selected pristine and -NH_2_-/-COOH-functionalized MNPs.

Based on the obtained results, the samples obtained at the flows of 30-30 and 50-30 were subjected to the subsequent advanced characterizations, as they showed the most uniform trends of increasing/decreasing the hydrodynamic diameter and zeta potential with increasing amounts of the functionalization agent (Figure 1).

Thus, the selected samples (30-30 and 50-30), both pristine and functionalized with 1, 3, and 5% -NH_2_/-COOH, were further characterized by XRD coupled with Rietveld refinement, SAED, TEM, HR-TEM, FT-IR, TG-DSC, and VSM.

**Figure 2:**
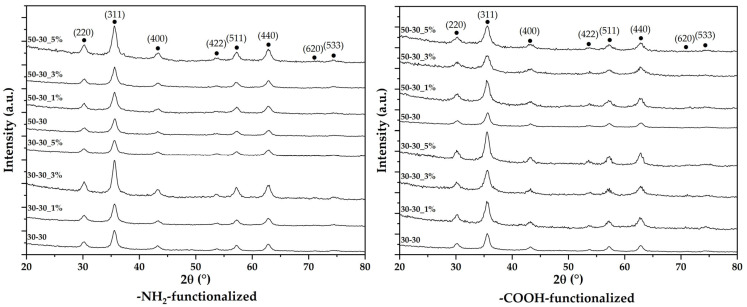
The diffractograms acquired for the pristine and -NH_2_-/-COOH-functionalized MNPs.

The XRD analysis was used to identify the crystalline phases present, while the Rietveld refinement allowed for determining the unit cell parameters, the average crystallite size, and the crystallinity of the samples. The diffractograms demonstrate the presence of a unique crystalline phase, with the diffraction interferences and the corresponding Miller indices characteristic to magnetite in the cubic crystallization system and the Fd3m space group for all the synthesized nanoparticles (according to JCPDS file 00-019-0629 [36,37]) (Figure 2).

The results obtained after Rietveld refinement are presented in Table 2. As it can be observed, higher flows decrease the cell unit, the average crystallite size, and the crystallinity of the nanoparticles. This effect could be explained by the formation of nucleation centers with lower concentrations of the precursors and, consequently, decreased nucleation rates when the co-precipitation occurs [38]. In this context, it is expected that the average nanoparticle size would be smaller in the case of the 50-30 samples. Furthermore, the presence of the functionalization agent generally increases the cell unit and decreases the average crystallite size. For the -NH_2_-functionalized nanoparticles, the crystallinity is lower than for the pristine nanoparticles, except for the 30-30_1% sample. By contrast, the crystallinity of the -COOH-functionalized nanoparticles is higher, except for the 50-30_3% sample.

**Figure 3:**
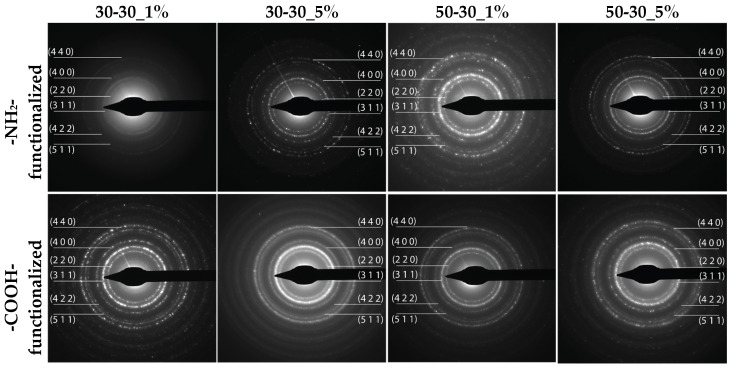
The SAED patterns for the 1 and 5% -NH_2_-/-COOH-functionalized MNPs.

Subsequently, the diffraction rings within the SAED patterns are associated with the Miller indices characteristic to magnetite, thus confirming its formation as a single crystalline phase (Figure 3). Additionally, the well-defined diffraction rings further demonstrate the high degree of crystallinity of the nanoparticles [1,39,40,41]. Thus, the previously obtained results are further confirmed.

**Figure 4:**
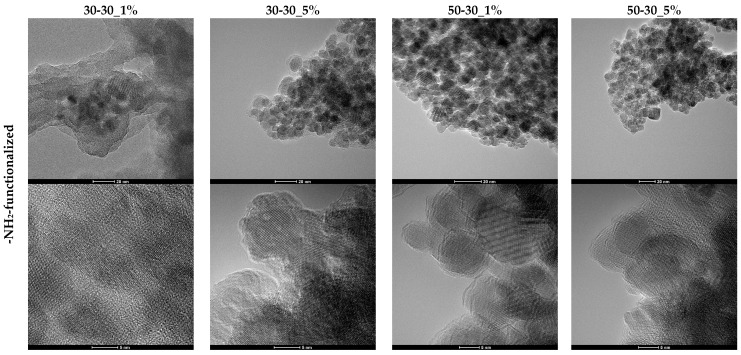

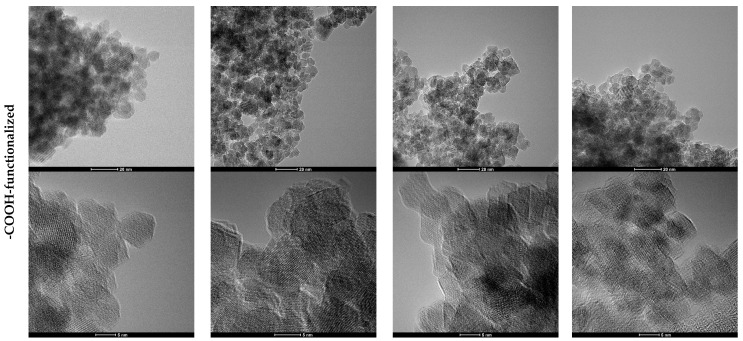
TEM and HR-TEM images for the 1 and 5% -NH_2_-/-COOH-functionalized MNPs.

Moreover, the nanoparticles were subjected to TEM and HR-TEM analysis in order to determine the morphology and the size of the nanoparticles (Figure 4). TEM and HR-TEM images demonstrate the formation of MNPs characterized by an increased tendency of agglomeration, due to the considerably reduced particle size. However, it can be observed that while the -NH_2_-functionalized nanoparticles are uniform and quasi-spherical, the addition of the -COOH functionalization agent also led to the formation of polyhedral nanoparticles, especially at higher flows and concentrations. Thus, it can be assumed that the functionalization agent also plays the role of a surfactant that determines the shape of the nanoparticles. Overall, the growth and coarsening of the nucleation centers and, subsequently, of the magnetite crystals have resulted in the formation of monocrystalline nanoparticles. Additionally, the HR-TEM images confirm both the crystallinity of the nanoparticles through the arrangement of the atomic planes and the presence of the organic component on the surface of the nanoparticles through the thin layer surrounding the magnetic core.

**Figure 5:**
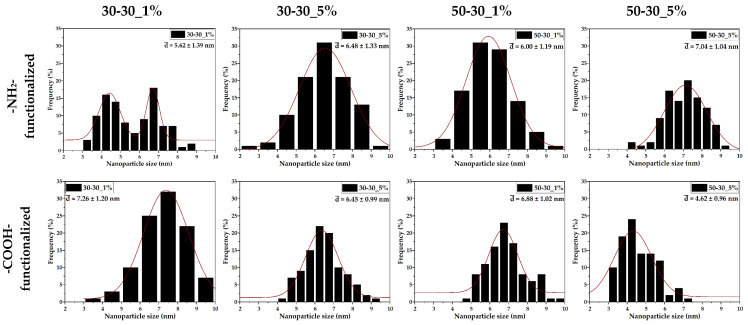
Size distributions and curve fittings for the 1 and 5% -NH_2_-/-COOH-functionalized MNPs.

The size distributions (Figure 5) are significantly narrow, with nanoparticle sizes ranging between 2 and 10 nm. Except for the -NH_2_-functionalized 30-30 sample, all distributions are monomodal, thus demonstrating the efficiency of microfluidic methods for ensuring nanoparticle size uniformity. Moreover, the average nanoparticle size is directly influenced by the presence and type of the functionalization agent and by the flow. Specifically, for the -NH_2_-functionalized nanoparticles, the average size increases with the concentration of the functionalization agent and the flow. By contrast, higher flows and SBA concentrations lead to decreases in the average size of the -COOH-functionalized nanoparticles. Additionally, since the average nanoparticle size varies between 4.5 and 7.5 nm, it can be concluded that the nanoparticles are monocrystalline.

The difference between the values obtained through DLS and TEM/HR-TEM is quite evident. It confirms the high degree of interaction between the functional groups present on the surface of the nanoparticles and the solvent molecules, which form an electric dipole layer surrounding the nanoparticle [42], as well as the high agglomeration tendency of the nanoparticles due to their significantly reduced sizes.

**Figure 6:**
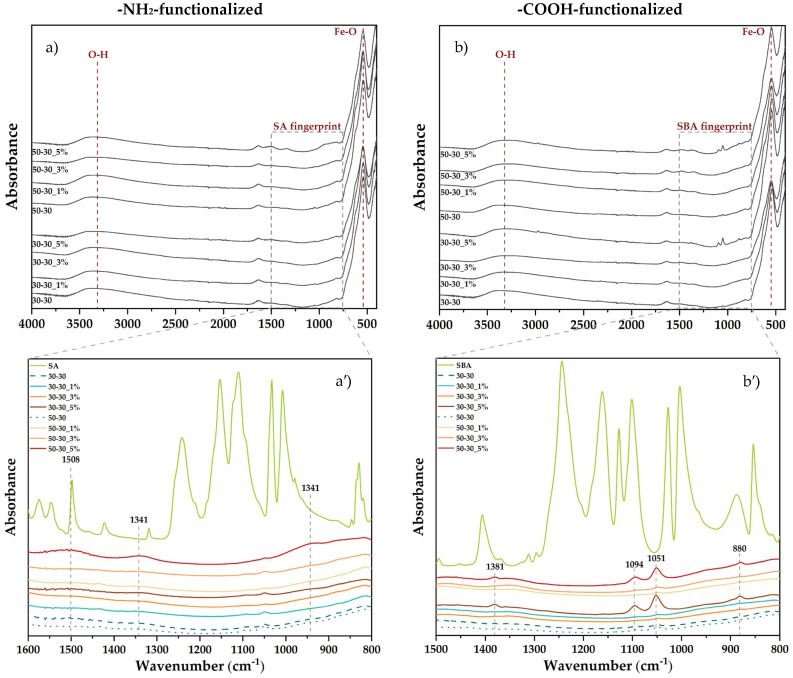
FT-IR spectra acquired for the pristine and -NH_2_-/-COOH-functionalized MNPs (**a**) and (**b**), full spectra, (**a′**) and (**b′**), marked area.

The absorption bands specific for the functional groups present within the pristine and -NH_2_-/-COOH-functionalized MNPs were assessed through FT-IR analysis (Figure 6). The absorption band specific to the Fe-O bond (540–545 cm^−1^) is present within all samples, thus confirming the formation of iron oxide. In addition, changes in the FTIR spectra due to the functionalization with SA (923, 1341, and 1508 cm^−1^) and SBA (880, 1051, 1094, 1381, and 2974 cm^−1^) can be observed at 5% concentrations, except for the 30-30 -NH_2_-functionalized sample. The low functionalization yield in this case could be caused by a reduced flow rate of the precipitation solution containing the functionalization agent and, consequently, a reduced interaction with the latter. The absorption bands at ~3300 cm^−1^ are characteristic of the O-H functional group.

Subsequently, the functionalization degree was assessed through TG-DSC (Figure S7) by recording the mass loss with increasing temperature. In the case of the 30-30 -NH_2_-functionalized samples, there are no significant differences, which demonstrates a low functionalization yield, and thus confirms the results obtained by FT-IR analysis. For the 50-30 -NH_2_-functionalized samples, the highest mass loss was recorded at the 5% concentration, with no major differences between the other three, which also confirms the FT-IR results. In the case of the -COOH-functionalized samples, the mass loss increases with the amount of the functionalization agent, except for the 30-30 5% sample, for which the lowest mass loss was recorded.

**Figure 7:**
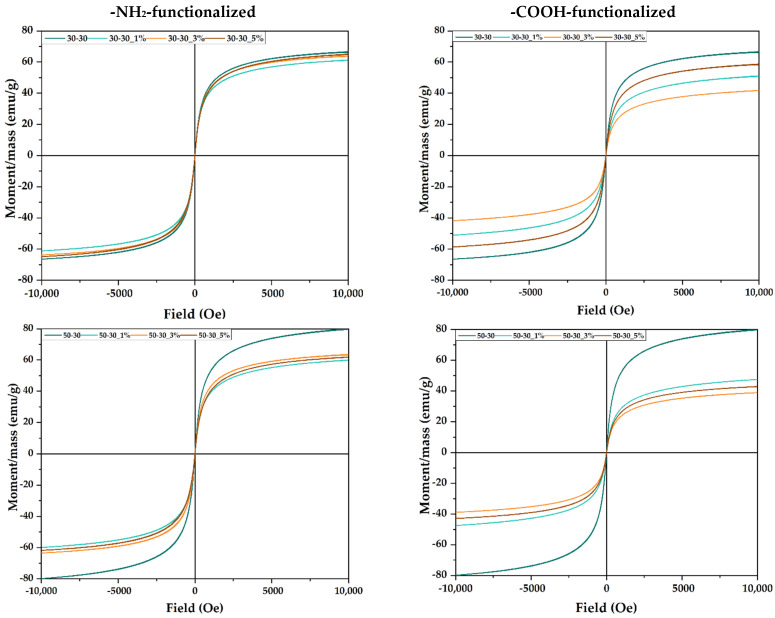
Field-dependent magnetization measurements for the pristine and -NH_2_-/-COOH-functionalized MNPs.

The magnetic properties of the nanoparticles were evaluated by VSM analysis (Figure 7). It can be observed that no sample shows hysteresis, thus confirming the formation of MNPs with superparamagnetic properties. Additionally, for the -NH_2_-functionalized samples, higher saturation magnetization values of approximately 60 emu/g were obtained, which is due to the larger size of the nanoparticles, according to the TEM analysis. Furthermore, for the 30-30 -NH_2_-functionalized sample, there are no significant differences between the pristine and the functionalized nanoparticles, which further confirms the low functionalization yield. However, for the other samples, the addition of the functionalization agent leads to a magnetization decrease, especially in the 50-30 -COOH-functionalized sample. Nonetheless, the magnetization values are similar to other studies that investigated MNPs with the same sizes [1,43].

## 4. Discussion

MNPs have been widely used in the past few years as nanostructured carriers for the controlled delivery and release of various bioactive molecules [6,44,45]. For this purpose, the nanoparticles should be synthesized in such a way that it allows for the control of their properties, especially in terms of size, shape, and surface reactivity. Such properties directly influence the behavior of the drug delivery systems inside the organism, namely their bioavailability, biodistribution, pharmacokinetics and pharmacodynamics, and body clearance [46,47]. Additionally, the surface of the nanoparticles should also ensure the possibility to conjugate the desired biomolecules that could further govern their release at the targeted site [48]. In this context, the choice of the synthesis method is fundamental for their final application.

Therefore, the aim of the present study was to develop a series of MNPs functionalized with amino and carboxyl groups through a microfluidic method. The approach involved the co-precipitation of the iron ions at the junction with the alkaline solution at the microchannel level. In this manner, by varying the synthesis parameters, improved control of the nanoparticle properties could be achieved. Specifically, the flows of the precursor and precipitation/functionalization solutions were varied in order to optimize the final properties of the nanoparticles. The mechanism of nanoparticle formation is based on the co-precipitation reactions, namely the simultaneous occurrence of nucleation, growth, coarsening and/or agglomeration processes [49]. In the context of microfluidic syntheses, the processes involved are significantly more controlled, since the maximum reaction environment measures 100 µm. Thus, the nucleation centers and, subsequently, the nanoparticles that are formed have narrow size distributions.

Although it is a relatively novel field, microfluidics has brought important potential in a variety of domains, especially within the pharmaceutical industry [50]. In this context, microfluidic synthesis methods have been extensively applied for achieving high control over the final characteristics of the nanomaterials that can be further used for diagnostic and therapeutical applications [50,51,52].

On the one hand, the geometry of the microfluidic platform and the flow and concentration of the reagent solutions have a direct implication in the morphology of the nanoparticles [53], with many studies reporting the synthesis of spherical [1,24,54,55,56,57], cubical [58], star-shaped [54,59,60,61], rod-like [58,62], or plate-shaped [63] nanoparticles. The parameters involved in the present microfluidic approach have led to the development of both spherical, which is the most common morphology obtained for MNPs [1,24,51], and polyhedral nanoparticles. Thus, this study demonstrates the possibility to control the shape of the obtained nanoparticles through the type and concentration of the functionalization agent used and the flow within the LoC device.

On the other hand, the geometry, flow, and concentrations can also influence the nanoparticle size and size distribution, which represent key features for drug delivery systems. The obtained results demonstrate a significantly narrow size distribution, which is similar [1,21,51] or even lower [24] than previously reported studies. Nonetheless, the size distributions are significantly narrower than those obtained through the conventional co-precipitation method. Thus, the efficiency of the microfluidic approach for obtaining highly uniform nanoparticles has been successfully demonstrated.

To the best of our knowledge, this study represents the first one-step synthesis of NH_2_-/-COOH-functionalized MNPs using a microfluidic approach. Generally, the functionalization step is performed post-synthesis [24], which does not ensure a high reaction yield. In this context, the degree of functionalization can be tuned through the amount of the functionalization agent added to the reaction.

Moreover, since both SA and SBA have been previously used as linkers between nanoparticles and bioactive molecules [64,65,66,67], they are safe to be used as functionalization agents for biomedical applications. Thus, the nanoparticles developed within the present study hold great potential for the attachment of bioactive molecules and their subsequent use as drug delivery systems. In this context, the magnetic properties of the nanoparticles are suitable for applications involving hyperthermia, magnetically directed drug release, or magnetoresistive sensor-based diagnostics [68,69,70].

## 5. Conclusions

The purpose of the present study was to investigate the influence of the reagent solution flows and the concentration of the functionalization agent on the final properties of MNPs. DLS and zeta potential measurements demonstrated optimal flows at 50 and 30 rpm for the precipitating/functionalization solution and 30 rpm for the precursor solution. The XRD and SAED results proved the successful synthesis of magnetite as the unique mineralogical phase within all cases. Furthermore, morphological investigations through TEM and HR-TEM showed significantly narrow distribution sizes and the possibility to obtain both spherical and polyhedral nanoparticles, depending on the type of the functionalization agent and the flows within the microfluidic platform. The optimal concentration of the functionalization agent should be 5%, in order to ensure a higher reaction yield. Finally, the magnetic properties of the systems are similar to those obtained for nanoparticles with considerably larger sizes. Thus, the potential of the present microfluidic platform for obtaining NH_2_-/-COOH-functionalized MNPs has been demonstrated.

## Figures and Tables

**Table 1:** The flows used for the synthesis of pristine and -NH_2_-/-COOH-functionalized MNPs.

Sample	30-10	30-15	30-20	30-25	30-30	50-10	50-20	50-30	50-40	50-50
Precipitating/functionalization solution flow (rpm)	30	30	30	30	30	50	50	50	50	50
Precursor solution flow (rpm)	10	15	20	25	30	10	20	30	40	50

**Table 2:** The unit cell parameters, average crystallite size, and crystallinity of the obtained MNPs.

Sample		Unit Cell Parameters						Average Crystallite Size (nm)	Crystallinity (%)
		a (Å)	b (Å)	c (Å)	α (°)	β (°)	γ (°)		
**Pristine**	30-30	8.355	8.355	8.355	90	90	90	8.06	18.53
	50-30	8.350	8.350	8.350	90	90	90	7.44	15.96
**-NH_2_-** **functionalized**	30-30_1%	8.356	8.356	8.356	90	90	90	7.09	19.95
	30-30_3%	8.360	8.360	8.360	90	90	90	8.10	16.74
	30-30_5%	8.350	8.350	8.350	90	90	90	7.93	17.33
	50-30_1%	8.355	8.355	8.355	90	90	90	7.06	17.26
	50-30_3%	8.357	8.357	8.357	90	90	90	7.60	20.83
	50-30_5%	8.356	8.356	8.356	90	90	90	7.04	18.37
**-COOH-** **functionalized**	30-30_1%	8.362	8.362	8.362	90	90	90	6.30	18.48
	30-30_3%	8.352	8.352	8.352	90	90	90	5.55	17.10
	30-30_5%	8.358	8.358	8.358	90	90	90	7.82	17.37
	50-30_1%	8.357	8.357	8.357	90	90	90	6.05	17.40
	50-30_3%	8.338	8.338	8.338	90	90	90	5.20	14.43
	50-30_5%	8.358	8.358	8.358	90	90	90	5.53	15.65

## Data Availability

Not applicable.

## Supplementary Materials

The following supporting information can be downloaded at: https://www.mdpi.com/article/10.3390/nano12183160/s1, Figure S1: The design of the LoC device; Figure S2: The geometry of the channel within the middle layer. Adapted from an open-access source [1]; Figure S3: The hydrodynamic diameter values of the pristine and -NH_2_-functionalized MNPs; Figure S4: The hydrodynamic diameter values of the pristine and -COOH-functionalized MNPs; Figure S5: The zeta potential values of the pristine and -NH2-functionalized MNPs; Figure S6: The zeta potential values of the pristine and -COOH-functionalized MNPs; Figure S7: TG-DSC curves for the pristine and -NH2-/-COOH-functionalized MNPs.
