# Supplementary material for: Microfluidic Synthesis of -NH2- and -COOH-Functionalized Magnetite Nanoparticles
Source: Nanomaterials (Basel). 2022 Sep 12;12(18):3160. doi: 10.3390/nano12183160 (PMC9503789; doi:10.3390/nano12183160)
Supplement: Supplementary file 1 [file nanomaterials-12-03160-s001.zip › nanomaterials-1886642-supplementary.pdf]

nanomaterials

Supplementary Materials

Microfluidic Synthesis of -NH2- and -COOH-Functionalized Magnetite Nanoparticles

Cristina Chircov [1,2], Alexandra Cătălina Bîrcă [1,2], Bogdan Stefan Vasile [1,2,3], Ovidiu-Cristian Oprea [2,4], Keng-Shiang Huang [5] and Alexandru Mihai Grumezescu [1,2,6,7,*]

1 Department of Science and Engineering of Oxide Materials and Nanomaterials, University Politehnica of Bucharest, 011061 Bucharest, Romania
2 National Research Center for Micro and Nanomaterials, University Politehnica of Bucharest, 060042 Bucharest, Romania
3 National Research Center for Food Safety, University Politehnica of Bucharest, 060042 Bucharest, Romania
4 Department of Inorganic Chemistry, Physical Chemistry and Electrochemistry, University Politehnica of Bucharest, 1-7 Polizu St., 011061 Bucharest, Romania
5 The School of Chinese Medicine for Post-Baccalaureate, I-Shou University, Kaohsiung 840301, Taiwan
6 Research Institute of the University of Bucharest—ICUB, University of Bucharest, 050657 Bucharest, Romania
7 Academy of Romanian Scientists, 54 Spl. Independentei, 050045 Bucharest, Romania
* Correspondence: grumezescu@yahoo.com

Figures S1 and S2 represent the schematic configuration of the microfluidic devices used for the synthesis of magnetite nanoparticles.

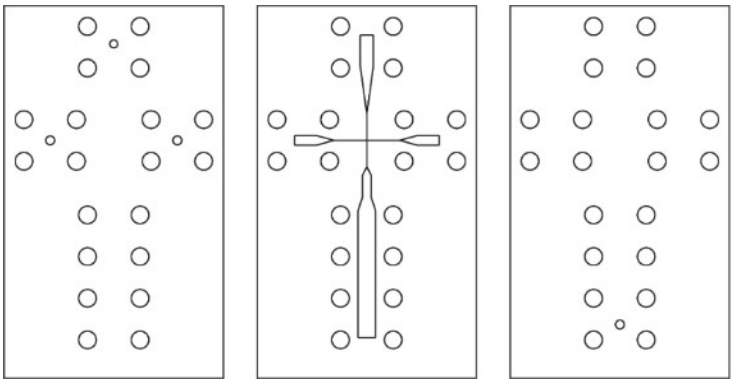

Figure S1. The design of the LoC device.

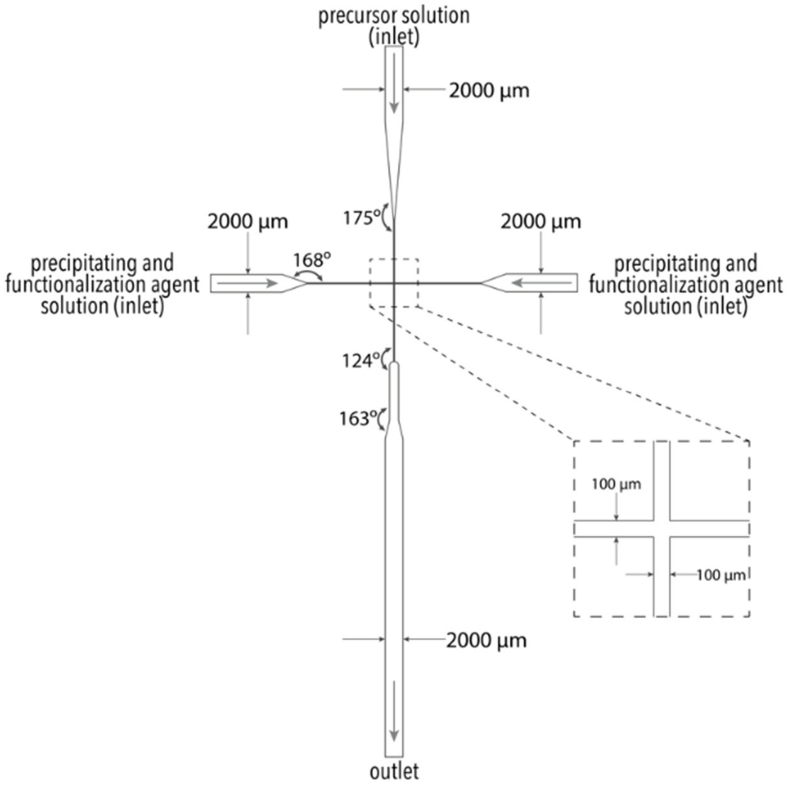

Figure S2. The geometry of the channel within the middle layer. Adapted from an open-access source [1].

Figures S3 and S4 show the values of the hydrodynamic diameter obtained for all the synthesized MNPs. Figures S3 and S4 show the values of the zeta potential obtained for all the synthesized MNPs.

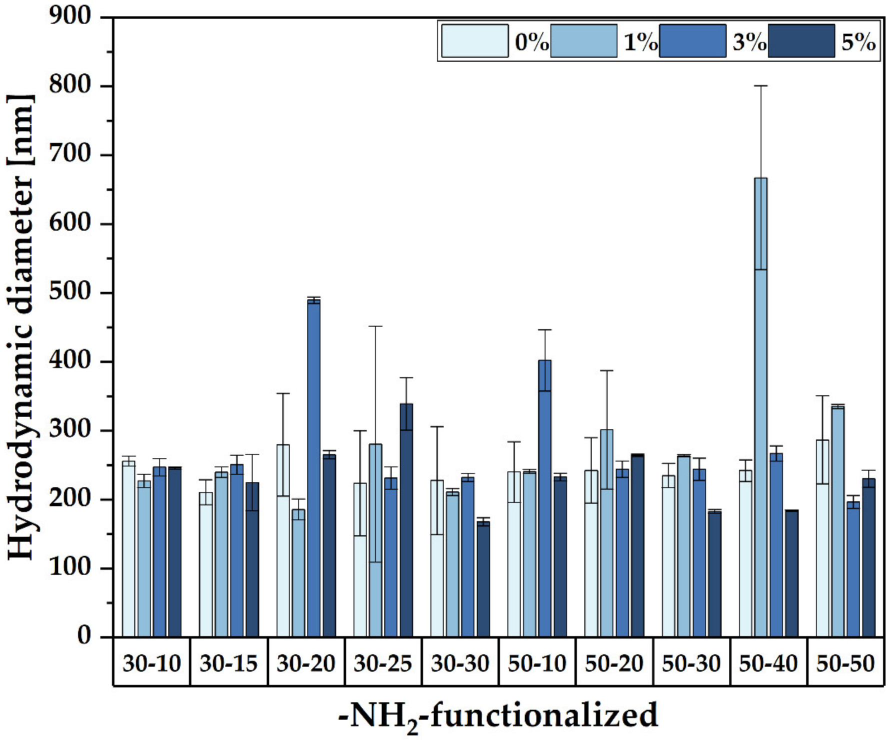

Figure S3. The hydrodynamic diameter values of the pristine and -NH$_2$-functionalized MNPs.

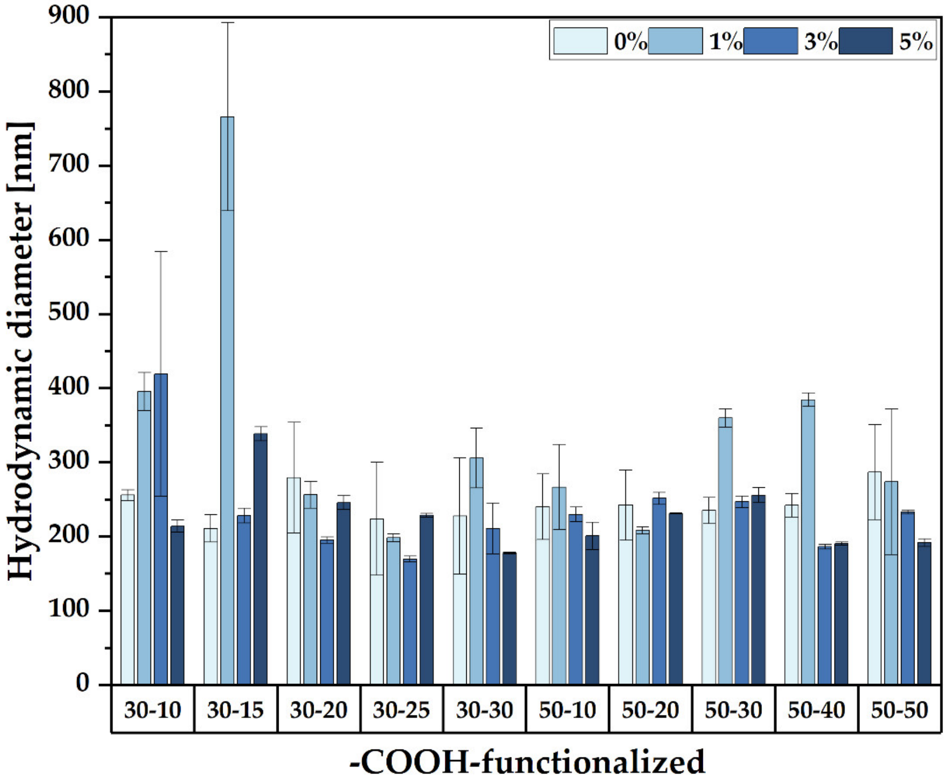

Figure S4. The hydrodynamic diameter values of the pristine and -COOH-functionalized MNPs.

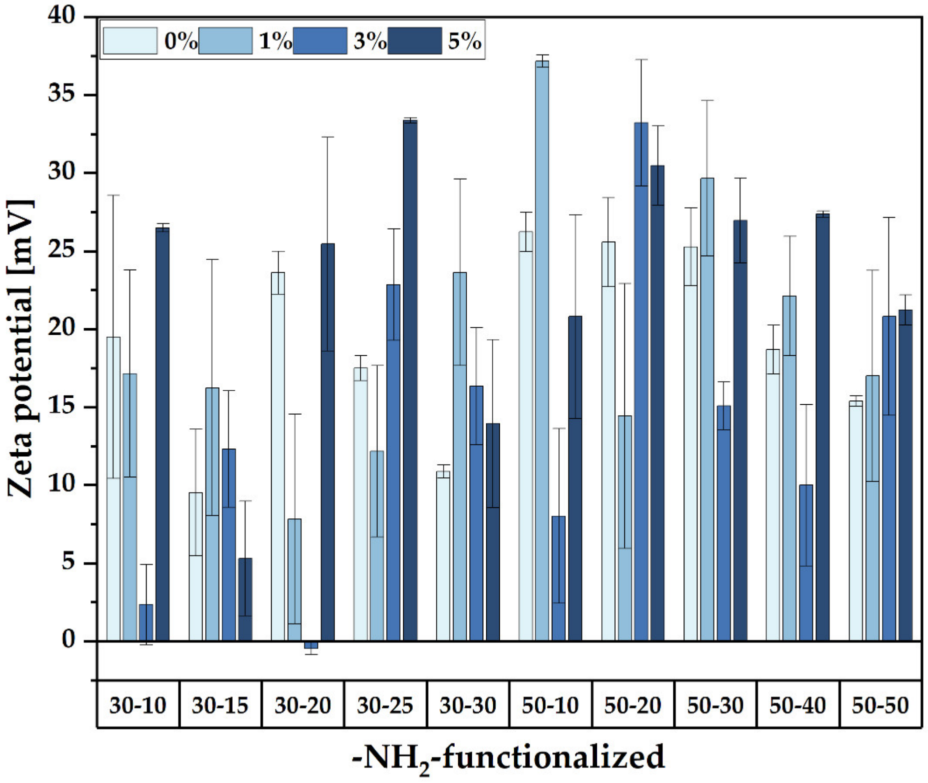

Figure S5. The zeta potential values of the pristine and -NH_2-functionalized MNPs.

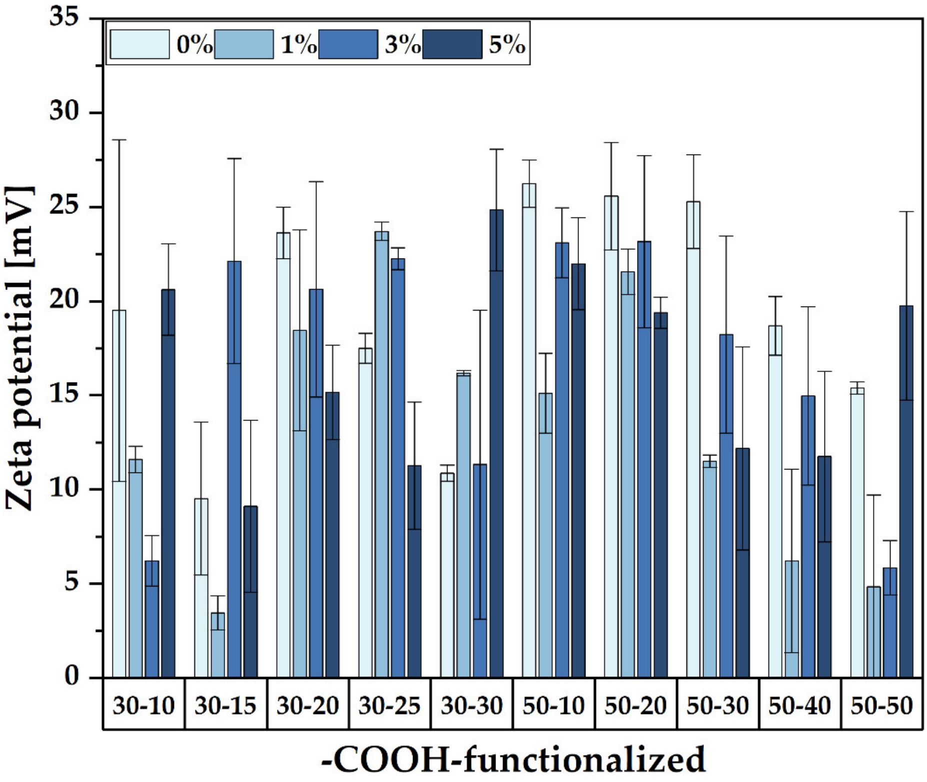

Figure S6. The zeta potential values of the pristine and -COOH-functionalized MNPs.

Figure S7 shows the TG-DSC curves for all the synthesized MNPs.

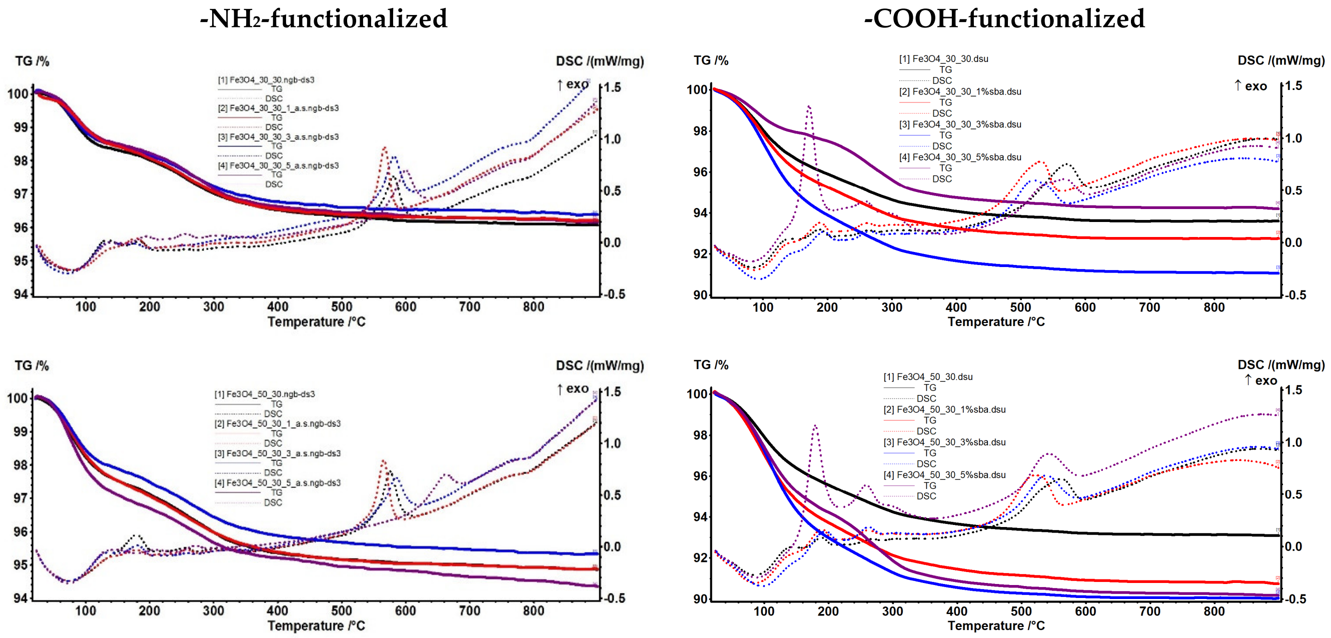

Figure S7. TG-DSC curves for the pristine and -NH_2-/-COOH-functionalized MNPs.

References

1. Chircov, C.; Bîrcă, A.C.; Grumezescu, A.M.; Vasile, B.S.; Oprea, O.; Nicoară, A.I.; Yang, C.-H.; Huang, K.-S.; Andronescu, E. Synthesis of magnetite nanoparticles through a lab-on-chip device. *Materials* **2021**, *14*, 5906.
